# Potential applicability of cytokines as biomarkers of disease activity in rheumatoid arthritis: Enzyme-linked immunosorbent spot assay-based evaluation of TNF-α, IL-1β, IL-10 and IL-17A

**DOI:** 10.1371/journal.pone.0246111

**Published:** 2021-01-26

**Authors:** Keerthie Dissanayake, Chandrika Jayasinghe, Priyani Wanigasekara, Ajith Sominanda

**Affiliations:** 1 Department of Anatomy, Faculty of Medicine, University of Peradeniya, Kandy, Sri Lanka; 2 Department of Medicine, Faculty of Medicine, University of Peradeniya, Kandy, Sri Lanka; 3 Teaching Hospital, Peradeniya, Sri Lanka; 4 Rehabilitation Hospital, Digana, Sri Lanka; University of Texas Southwestern Medical Center, UNITED STATES

## Abstract

Biomarkers play a pivotal role in the management of rheumatoid arthritis (RA) by facilitating early diagnosis and ‘treat to the target.’ However, no gold standard biomarker has been identified for monitoring the disease activity in RA. Cytokines, a diverse group of small protein molecules secreted by peripheral blood mononuclear cells (PBMCs), play a pivotal role in pathogenesis and disease progression in RA. Research is currently underway to find out the applicability of cytokines as biomarkers in RA. This study aimed to quantify the PBMCs that secrete four types of cytokines; TNF-α, IL-1β, IL-10 and IL-17A in two cohorts of active RA patients (early RA patients and established RA patients), compared to healthy controls (HC), using the enzyme-linked immunosorbent spot (ELISPOT) assay, and to assess their association with measures of disease activity of RA. Patients were recruited from outpatient rheumatology clinics, and the disease activity was assessed using single and composite measures of disease activity. The cytokine expression was evaluated using freshly separated PBMCs from whole blood of RA patients using the ELISPOT assay. The number of PBMCs (counted as spot-forming cells (SFCs) per 10^5^ PBMCs) that secreted the cytokine of interest were statistically significantly higher in early RA patients, compared to HC, for IL-17A (P<0.05). Such an increased number of SFCs was not observed in the established RA group, compared to controls, for any of the cytokines tested. The correlation analysis showed that IL-17A is having a moderate correlation (Spearman`s ρ, p <0.05) with five clinical measures of disease activity, including disease activity score 28 (DAS28). According to the multivariable linear regression models, IL17A was a good predictor of both the disease activity score 28 (DAS28) and clinical disease activity index (CDAI). In conclusion, IL-17A has potential applicability as a biomarker of disease activity of RA.

## Introduction

Rheumatoid arthritis (RA) is a chronic systemic autoimmune joint disease that leads to joint inflammation, deformity, and loss of function [1]. With the advent of newer therapeutics, the outcome of RA seems to be improving in recent years. The recommendation of ‘treat to target’ strategy by the European League against Rheumatism (EULAR) and American College of Rheumatology (ACR) indicates the need for outcome-oriented therapeutic interventions. Evaluation of the disease activity of RA facilitates ‘treat to target’ strategy by enabling better treatment selection and dose adjustments [2, 3].

Biomarkers are chemical, physical, or biological measures that can be used in refining the diagnosis, measuring the disease progression, or predicting and monitoring the effects of treatment [4]. Antibodies to citrullinated peptide antigen (ACPA) [5], rheumatoid factor (RF), C-reactive protein (CRP), and fibrinogen levels (as measured by erythrocyte sedimentation rate (ESR)) are some of such measures. ACPA and RF are useful in terms of diagnosis and predicting disease progression. ESR and CRP are used as markers of disease activity in RA, though it may not accurately reflects the disease activity in RA [6]. In a study carried out by Kay et al., among 9,135 with active RA, 58% of patients had elevated levels of neither ESR nor CRP. Composite measures such as disease activity score 28 (DAS 28) and simplified disease activity index (SDAI) has integrated many dimensions of the disease to quantify disease activity of RA [7, 8]. A test involving the combination of 12 serum biomarkers, the multi-biomarker disease activity test (MBDA), is in wide use in research and clinical practice for assessing the disease activity in RA [9, 10]. However, none of the currently used biomarkers in RA seems to be the gold standard, especially in terms of therapeutic monitoring. Therefore, research in finding better biomarkers are continuing.

In an era of advanced molecular technologies, cytokines are considered as potential biomarkers [11]. Cytokines are a diverse group of small protein molecules secreted by a wide range of cells and have specific roles during cellular interactions and communications. Peripheral blood mononuclear cells (PBMCs) consisting of lymphocytes and monocytes, also produce cytokines [12]. They are synthesized ‘as needed’ during the immune response. An imbalance of the complex cytokine network can lead to inflammation and, in turn, tissue damage. Uncontrolled production of pro-inflammatory cytokines may promote autoimmunity and play a vital role in the development and perpetuation of RA [13].

The serum levels of cytokines and their association with disease activity of RA has been extensively studied [14–16]. A study carried out by Meyer P.W. et al. revealed that IFNγ, IL-1β, IL-1R, TNF-α, GM-CSF and VEGF significantly correlated with disease activity in patients with high disease activity (DAS28 > 5.1) [17]. The serum levels of IL-6, IL-10, and IFNγ have been correlated with radiological progression of RA [15]. However, some of the previous studies carried out to assess the relationship of serum cytokine levels and physical measures of disease activity in RA contradict each other for certain cytokines [18, 19] possibly due to different factors including, but not limited to, type of the bioassay used for cytokine detection and their sensitivity, patient characteristics, concomitant medications, sample handling and cytokine stability.

Among the many techniques available for cytokine studies, ELISA has been widely used [20]. However, ELISPOT assay is more sensitive compared to ELISA, thus, a better immunoassay for studying the cytokine expression during the immune response [21, 22]. This is specially applicable when studying the cytokines which are less abundant in serum. These two bioassays measure two dimensions of the cytokine expression. While ELISA quantifies the already secreted and biologically available cytokine levels in body fluids, ELISPOT measures the *in-vitro* secretion of cytokines by the committed PBMCs by quantifying the cell number. Furthermore, the ELISPOT assay can detect even a single cell that secretes the cytokine of interest from millions of PBMCs [23]. There are many other alternative methods for cytokine detection which were developed based on the technology of ELISA [24], flow cytometry [25], and chemiluminescence [26]. Each of these methods has its own advantages and disadvantages. Profiling cytokines and assessing their association with other biomarkers of RA could help to find cytokine-based biomarkers in RA. Furthermore, this would further expand the existing knowledge about these cytokines in RA. While evaluating the association with RA disease activity, ELISPOT based cytokine expression analysis would further explore the inconsistent results found in previous ELISA based cytokine studies.

This study aimed to assess the expression of TNF-α, IL-1β, IL-10 and IL-17A by PBMCs in two cohorts of active RA patients (DMARDS naïve, early RA patients and established RA patients who are on DMARDs), compared to healthy controls, using ELISPOT assay, and to assess their associations with clinical markers of RA disease activity and ESR.

## Materials and methods

### Patient recruitment

Ethical permission was obtained from the Institutional Ethical Review Committee, Faculty of Medicine, University of Peradeniya, Sri Lanka (2014/EC/65). Two cohorts of RA patients: DMARDs naïve early RA patients (disease duration < 6 months)(n = 12) and established RA patients on DMARDs (disease duration ≥ 6 months)(n = 17), attending two specialized Rheumatology clinics at Teaching Hospital, Peradeniya and Rehabilitation Hospital, Digana, in the central province of Sri Lanka were recruited to the study between June 2016—April 2017. The Consecutive and convenient sampling technique was used to recruit patients. Thus, due to the method used for patient recruitment and, certain demographic characteristics of the RA patients such as the high female preponderance of the disease, the recruited study sample was non-representative of a larger population.

Those who met the following inclusion and exclusion criteria were recruited to the respective groups, after obtaining the informed written consent.

#### DMARDs naïve early RA patients

Inclusion criteria,

- Age over 18 years
- RA diagnosed according to the 2010 ACR/EULAR classification criteria for RA
- RA with a duration of disease/symptoms of <6 months
- Rheumatoid factor (RF) positive
- Not exposed to any DMARDs previously
- Free from prednisolone or other steroids use for at least four weeks.

Exclusion criteria,

- The presence of other clinically significant disease conditions that can interfere with cytokine expressions such as acute or chronic infections

#### Established RA patients who are on DMARDs

Inclusion criteria,

- Age over 18 years
- RA with a duration of disease/symptoms of ≥ 6 months
- Rheumatoid factor (RF) positive
- Following up in the clinic with combined DMARDs including Methotrexate at stable doses for > 4 weeks
- Free from prednisolone or other steroids use for at least four weeks

Exclusion criteria for this cohort included

- The presence of a clinically significant comorbid condition that can interfere with cytokine expressions such as acute or chronic infection
- RA patients in disease remission (DAS 28 < 2.6)

In parallel, age and sex-matched healthy individuals (n = 11) were recruited, after obtaining the informed, written consent, as the control sample of the study.

### Collection of clinical and para-clinical data

Demographic data were collected by interviewing the patients. The disease activity of each of the patient was assessed with DAS28 and CDAI, and the joint pain was evaluated using pain-visual analog scale (0–10). DAS 28 was calculated by considering four parameters: tender joint count (0–28), swollen joint count (0–28), the patient’s global assessment of general health (0–10), and erythrocyte sedimentation rate (ESR). For calculating the DAS28, an online calculator (http://www.das-score.nl/das28/en) was used. CDAI was calculated by the summation of tender joint count (0–28), swollen joint count (0–28), patient global health assessment (0–10 visual analog scale), and physician global health assessment (0–10 visual analog scale).

### ELISPOT assay

Ready-made ELISPOT assay kits for TNF-α (ab46550, Abcam, UK), IL-1β (ab46578, Abcam, UK), IL-10 (ab46549, Abcam, UK) and IL-17A (ab83712, Abcam, UK) were purchased along with PVDF bottomed eight well-stripped microtitre plates (Merck Millipore, USA). The expression of all four cytokines of a given patient was tested simultaneously.

#### Preparation of PVDF bottomed microtitre plates for the assay

ELISPOT assays were performed according to the manufacturer’s instructions. In brief, PVDF bottomed eight well-stripped microtitre plates were primed by adding 25μL of 35% ethanol to each well and incubating at RT for 30 seconds. Wells were emptied from ethanol and washed thrice with 100 μl PBS. Then, 100 μl of diluted (1:100) capture antibodies of TNF-α, IL-1β, IL-10, and IL-17A were added to respective wells of the microtitre plates (4 wells for each cytokine per patient) and incubated at 4°C overnight. Following day, the capture antibodies were discarded, and the wells were washed once with PBS. Subsequently, the wells were blocked by adding 100 μl of blocking buffer (RPMI + 10% foetal bovine serum) to each well and incubating at room temperature (RT) for 2 hours. Then, the blocking buffer was discarded from wells of the microtitre plates and washed once with PBS.

#### Preparation of PBMCs for the assay

Venous blood (4 ml), from patients recruited in the study, were collected to EDTA tubes adhering to standard venipuncture techniques and universal precautions. PBMCs were separated from the whole blood, within 2 h of venepuncture, by density gradient centrifugation (Lymphoprep™, AXIS-SHIELD PoC AS, Norway). In brief, 4 ml of blood samples were diluted by adding an equal volume of 0.9% NaCl. The diluted blood samples were layered over 4 ml of Lymphoprep™ in a 15 ml conical centrifuge tubes. The tubes were centrifuged at 800 g for 20 minutes in a swing-out rotor at RT. After centrifugation, the distinct band formed at the sample/Lymphoprep medium interface (the PBMC layer) was removed using a pipette. Harvested PBMCs were diluted in 5 ml of 0.9% NaCl to reduce the density of the solution and was centrifuged for 10 minutes at 250 g at RT. The supernatant was decanted, and the formed cell pellet was re-suspended in 4 ml of complete media (RPMI supplemented with 10% foetal bovine serum and 1% Penicillin/Streptomycin). PBMC counting was done using the haemocytometer, and the percentages of viable PBMCs were determined using Trypan Blue dye exclusion method (T8154, Sigma-Aldrich, USA).

#### Adding PBMCs to microtiter plates

Freshly isolated PBMCs (as described previously) from whole blood of RA patients and HC were diluted appropriately for obtaining optimum cell numbers. These numbers of PBMCs added (in 100 μl) for TNF-α, IL-1β, IL-10, and IL-17A ELISPOT assays were 3125, 25000, 25000, and 100,000, respectively which were based on the ELISPOT kit manufacturer’s recommendations and prior optimizations.

Prepared PBMCs of a given patients sample was added to each of the 4 wells prepared for each cytokine. Of these four wells, two were supplemented with mitogen/s (to detect mitogen-induced cytokine expression) while the other two wells were supplemented with a similar volume of complete media (to detect spontaneous cytokine expression). In this regard, Lipopolysaccharide (L8274, Sigma-Aldrich, USA) was added at 1 μg/mL concentration to stimulate both TNF-α and IL-1β expression by PBMCs. Phorbol 12-myristate 13-acetate (PMA) (ab120297, Abcam, UK) and Ionomycin (ab120116, Abcam, UK) were added respectively at 1 ng/mL and 500 ng/mL concentrations to stimulate both IL-10 and IL-17A expression by PBMCs. Samples were incubated in a CO_2_ incubator at 37°C for 20 hours.

After the incubation, corresponding diluted biotinylated detection antibodies (1:100) were added, and the plates were further incubated at RT for 90 minutes. Then, the detection antibodies were discarded, and the wells were washed thrice with wash buffer (0.05% PBS-Tween). Subsequently, 100 μl of diluted Streptavidin-AP conjugate (1:1000) were added to each well, plates were covered, and incubated at RT for one hour. Then the plates were emptied, and the wells were washed thrice with wash buffer (0.05% PBS-Tween).

Then, 100 μl of 5-bromo-4-chloro-3-indolyl phosphate (*BCIP*)/nitro blue tetrazolium (*NBT*) buffer was added to each well, and the plates were incubated for 15 minutes. After removing BCIP/NBT, the wells were washed thrice with distilled water. Finally, the plates were dried, and the formed spots on the PVDF membrane of the plates were visualized. Each spot that developed on the PVDF membrane was representative of a PBMC that secreted the cytokine of interest. The number of PBMCs that secreted the cytokine of interest, i.e. spot-forming cells (SFC), was determined manually by counting the number of spots visible on the entire area of the PVDF membrane of each well, using a dissecting microscope. The average of the SFCs in two replicate wells for each sample was calculated, and adjusted for 10^5^ PBMCs.

### Statistical analysis

Continuous outcomes, including the number of spot forming cells (SFC) per 10^5^ PBMCs were reported as the median and interquartile range (IQR). Non-parametric statistics were used for the data analysis. Wilcoxon matched-pairs signed-rank test was used for comparing the SFCs based on mitogen stimulation. The numbers of cytokine specific SFCs per 10^5^ PBMCs were compared in the two experimental groups and the control group using the Kruskal-Wallis test. If significant, then Dunn’s multiple comparison test for post hoc correction was performed to account for multiple comparisons of each cytokine. It should be considered that this approach does not fully account for multiple testing as p-values were not adjusted across cytokines. Given the small number of cytokines tested, this is expected to only marginally increase the overall likelihood of false-positive results. Spearman’s rank correlation coefficient (ρ) was used to evaluate the correlation between multiple variables, including the number of SFCs/10^5^ PBMCs for each cytokine. The strength of the correlation was categorized according to correlation coefficient (ρ): 0.00–0.19 (very weak), 0.20–0.39 (weak), 0.40–0.59 (moderate), 0.60–0.79 (strong), 0.80–0.10 (very strong) [27]. Correlation of composite measures of disease activity, with cytokine expression and ESR, were also analyzed with multiple linear regression models. The number of the variables included in the models was based on the stepwise regression and the sample number. Stepwise regression was carried out in order to identify the subset of variables from the tested cytokines and ESR, resulting in the best performing model. As the dependant variable, both DAS 28 and CDAI were used separately in two models. However, ESR was not included as a predictor in the model where disease activity (dependent variable) was assessed with DAS 28 as it is an integral part of DAS 28 calculation. Linear regression assumptions were tested by creating diagnostic plots (Residuals vs. Fitted, Normal Q-Q., Scale-Location, Residuals vs. Leverage). For data analysis and visualization, R [28] and GraphPad Prism version 5.03 (GraphPad Software, San Diego, USA) were used. P < 0.05 were considered as statistically significant.

## Results

Altogether 29 RA patients and 11 healthy controls were recruited. Middle-aged female patients were predominant in both early and established RA patient cohorts. Demographic features, disease characteristics, and laboratory findings of the two RA patient groups and healthy controls (HC) are shown in Table 1.

**Table 1:** Demographic and disease characteristics of RA patients and HCs.

Characteristic	Early RA	Established RA	HC
	(n = 12)	(n = 17)	(n = 11)
Female (%)	83.33	88.23	72.72
Age, years	44.91(±10.07)	51.70(± 11.34)	46.9 (±14.73)
Disease duration, months,	4.75(±1.63)	37.05(±22.04)	-
Duration of current DMARDs regime (months)	-	18.64 (±13.98)	
Number of tender joints	7.66 (±3.59)	4.64 (± 5.72)	-
Number of swollen joints	2.58 (±2.0)	2.11 (±1.52)	-
PGH (0–100)	48.33 (±7.99)	48.82 (±16.04)	-
Joint pain- VAS (0–10)	5.08 (±1.03)	4.17 (±1.88)	-
ESR(mm 1^st^ hour)	49.33 (±19.14)	46.29 (±20.20)	-
DAS 28-ESR	5.27 (±0.56)	4.62 (±1.13)	-
CDAI	20(±5.16)	14.35(±9.41)	

Mean are shown with standard deviation (within brackets) unless specified. Disease-modifying antirheumatic drugs (DMARD); PGH–patient global health; VAS–visual analog scale; ESR–erythrocyte sedimentation rate; CDAI-clinical disease activity index, RA-rheumatoid arthritis, HC-healthy controls

**Fig 1:**
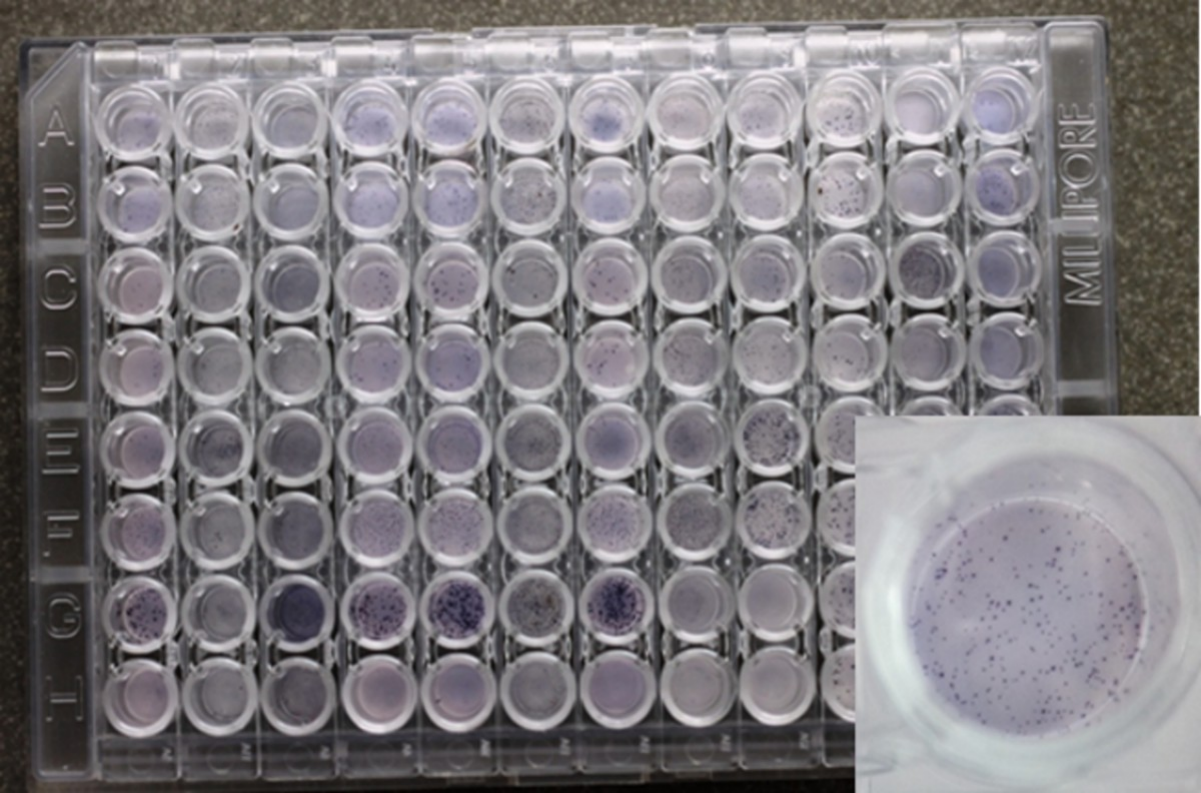
Representative image of PVDF bottomed ELISPOT plates. One well is magnified to illustrate the appearance of the spot forming cells (SFCs) on the PVDF membrane. SFCs were counted using a dissection microscope.

The expression of cytokine was indirectly quantified by assessing the number of PBMCs secreting the cytokine of interest, i.e., spot forming cells (SFCs) per 10^5^ PBMCs. For each biological sample, two technical replicates under mitogen-stimulated conditions and non-stimulated conditions were assessed (Fig 1).

Table 2 shows the median (inter-quartile range) spot-forming cells (SFCs) per 10^5^ PBMCs of 2 RA patient groups and HCs with and without mitogen stimulation. The number of SFC for all 4 cytokines were higher in mitogen-stimulated condition compared to non-stimulated conditions (P<0.05) except in IL-17A SFCs in early RA. The numbers of IL-10 SFCs were negligible in all 3 study groups in the absence of mitogen stimulation. Therefore, subsequent data analysis was based on the cytokine expression under mitogen-stimulated conditions.

**Table 2:** Median and inter-quartile ranges of Spot Forming Cells (SFCs) per 10^5^ PBMCs in the presence or absence of mitogen stimulation of PBMCs in RA patient groups and HCs.

	Mitogen stimulated	Non stimulated	p value
**TNF-α**			
Early RA (ERA)	14520 (11940–19533)	11560 (10273–15262)	p = 0.0025
Established RA (EsRA)	9057(3756–11311)	6733(1559–8993)	p = 0.0003
Healthy Controls	6200(4566–12800)	5133(3433–10888)	p = 0.0010
**IL-1β**			
Early RA (ERA)	7000(2594–10434)	5858 (1685–8877)	p = 0.0025
Established RA (EsRA)	2622(1008–5238)	2044(720–4138)	p = 0.0003
Healthy Controls	2700(1911–3828)	1866 (1266–2800)	p = 0.0010
**IL-17A**			
Early RA (ERA)	653 (266–758)	554(240–873)	p = 0.9063
Established RA (EsRA)	348(185–810)	165(88–546)	p = 0.0138
Healthy Controls	244(124–533)	133(49–205)	p = 0.0038
**IL-10**			
Early RA (ERA)	185 (133–213)	1 (1–3)	p = 0.0025
Established RA (EsRA)	121(20–201)	1(1–3)	p = 0.0003
Healthy Controls	82(39–119)	2(1–2)	p = 0.0010

Test of statistic–Wilcoxon matched-pairs signed-rank test, p <0.05 is considered statistically significant. PBMCs; peripheral blood mononuclear cells.

**Fig 2:**
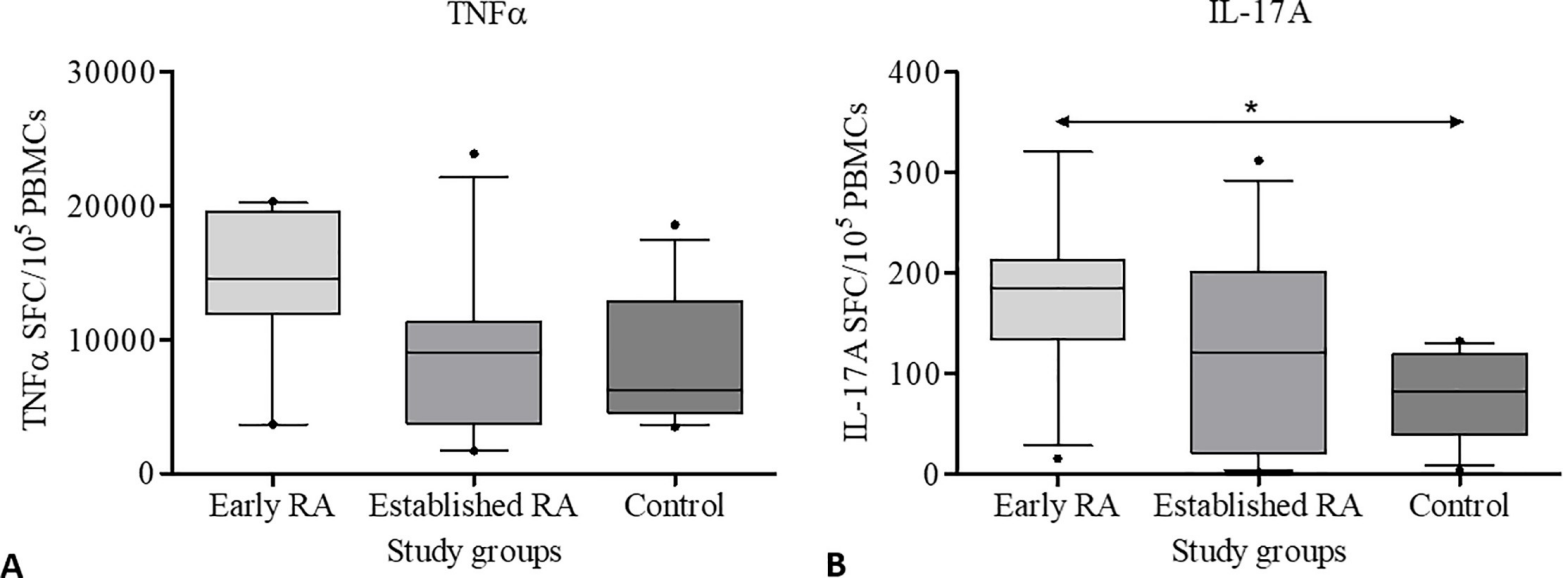
Box and Whiskers plot illustrating the mitogen-stimulated expression of cytokines by PBMCs of early RA patients, established RA patients, and controls. **A.** TNF-α (p = 0.0282), **B.** IL-17A (p = 0.0216). Error bars indicate 10–90 percentiles. P < 0.05 was considered statistically significant. SFCs- spot forming cells, PBMC- peripheral blood mononuclear cells. Statistical test–Kruskal-Wallis test. An asterisk indicates the statistically significant difference between the two groups.

Using the Kruskal-Wallis test, median numbers of SFCs were compared in the 3 study groups (early RA, established RA, and control) for all 4 cytokines separately. Regarding TNF-α and IL17A, median numbers of SFC per 10^5^ PBMC were different among patients in 3 study groups (p = 0.0282 and p = 0.0216, respectively). Despite such overall differences were observed, further analysis by Dunn’s multiple comparison test did not show a difference between any of the compared groups (early RA vs control, established RA vs control, and early RA vs established RA) for TNF-α (Fig 2). However, for IL-17A, the median number of SFC per 10^5^ PBMC was higher in early RA compared to the control according to Dunn’s multiple comparison test. Concerning IL10 and IL-1β, the median numbers of SFCs were not different among the study groups.

**Fig 3:**
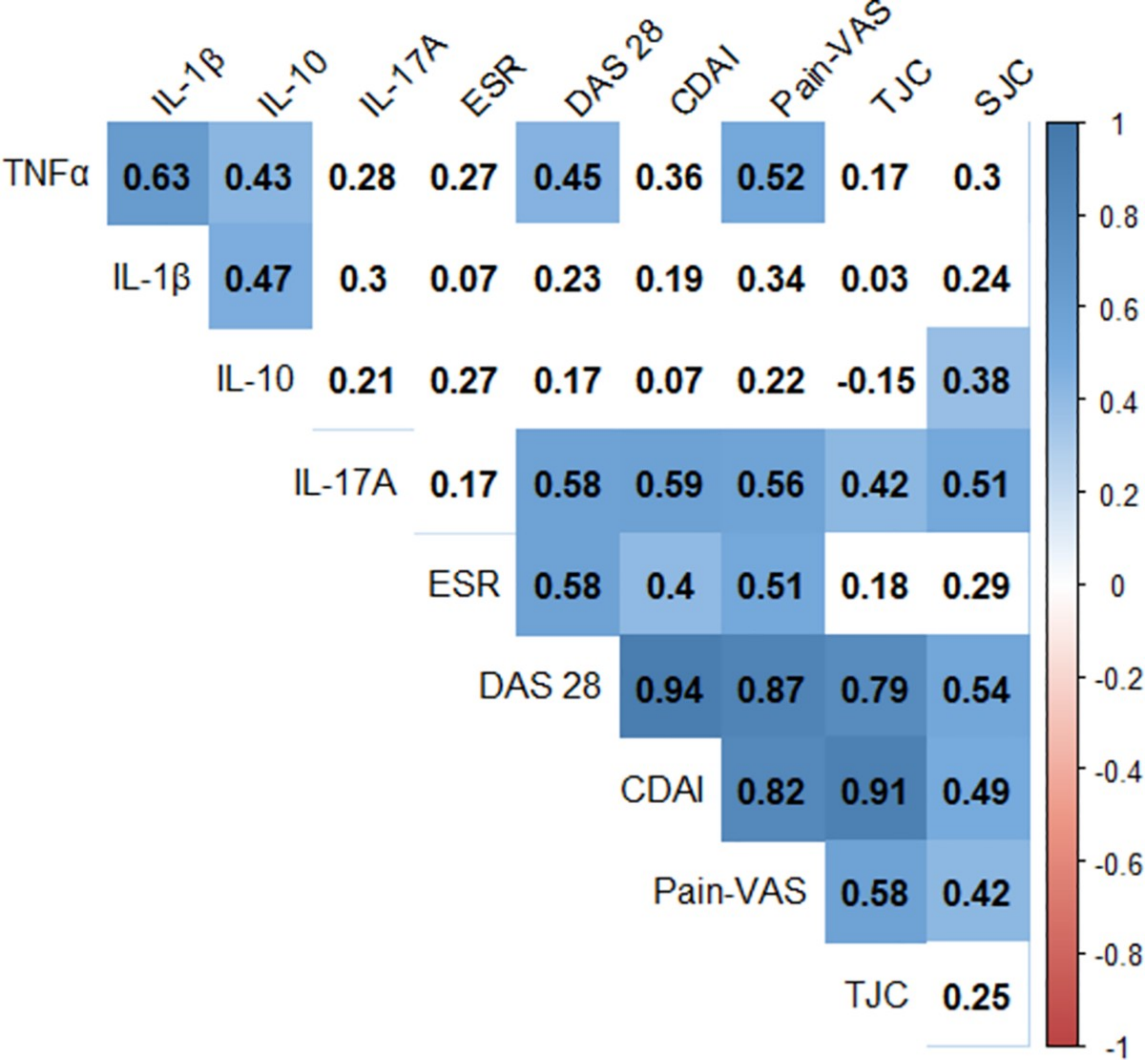
Correlogram illustrating the correlation coefficient between different measures of disease activity. The four cytokines tested along with the single and composite measures of disease activity have been included in the correlation matrix. The colour scale visualizes the strength and direction of the correlation. Numbers indicate the Spearman’s correlation coefficient (ρ) and only the numbers on blue background are statistically significant (p<0.05). Statistical test- Spearman’s rank correlation coefficient (ρ). The strength of correlation ‘ρ’: 0.00–0.19 (very weak), 0.20–0.39 (weak), 0.40–0.59 (moderate), 0.60–0.79 (strong), 0.80–0.10 (very strong). ESR-erythrocyte sedimentation rate, TJC-tender joint count, SJC-swollen joint count, Pain-VAS- joint pain as measured by the visual analog scale, DAS 28- disease activity score 28, CDAI-clinical disease activity index.

A correlation matrix was plotted to visualize the number of SFCs referring to four cytokines of interest (TNF-α, IL-1β, IL-17A, IL-10) and single measures of disease activity (ESR, joint pain-visual analog scale, swollen join count, tender joint count) and composite measures of disease activity (DAS 28 and CDAI) in all RA patients together (n = 29) (Fig 3). Cytokines that showed moderate strength of correlations with DAS 28 score were IL-17A (ρ = 0.58) and TNF-α (ρ = 0.45). IL-17A SFCs had a moderate correlation with CDAI (ρ = 0.59). Moreover, IL17A SFCs showed a moderate correlation with join pain-VAS (ρ = 0.59) in addition to swollen and tender joint counts. TNF-α SFCs had moderate correlations with DAS28 and joint pain-VAS. While IL10 SFCs had a moderate correlation with the swollen joint count, the number of IL1β SFCs were not correlated with any of the clinical markers of disease activity. None of the cytokines showed a strong correlation with CDAI or DAS 28.

**Fig 4:**
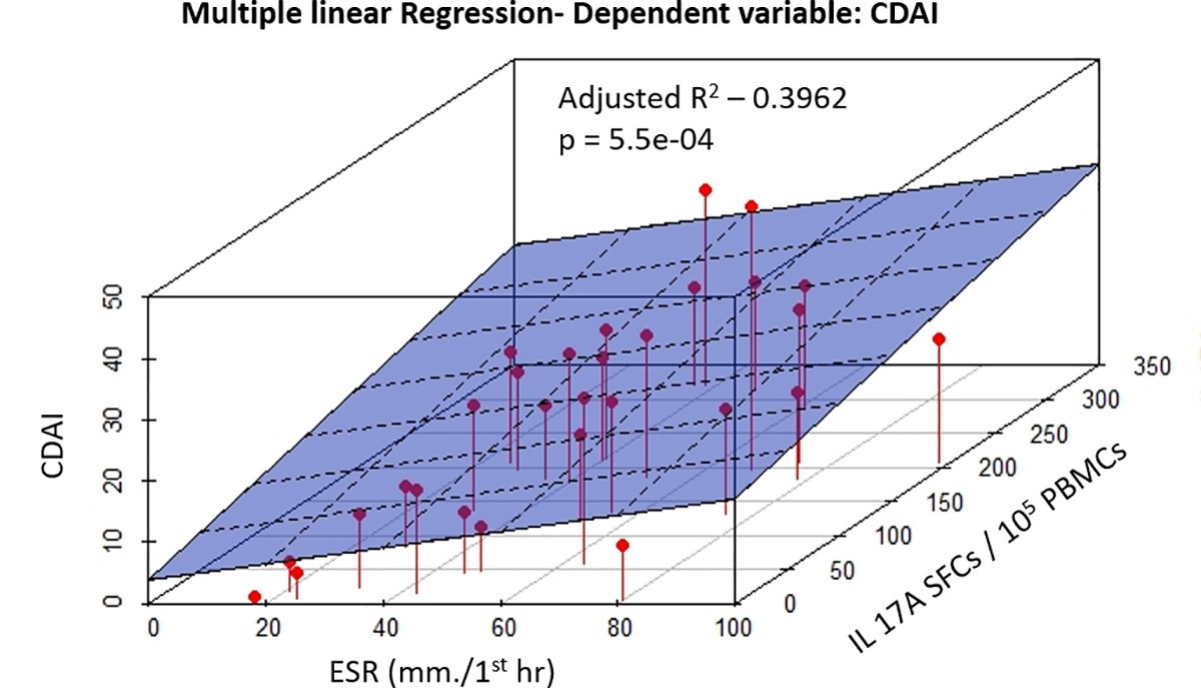
3D linear regression plot visualizes correlations between the CDAI, ESR and IL17A.

The association of composite measures of disease activity, as assessed by DAS 28 and CDAI, with the number of SFCs for different cytokines and ESR were further analyzed by multiple linear regression (Table 3). Based on the stepwise model selection, the best subset of covariates were identified for model 1 (DAS 28 as the dependent variable) and model 2 (CDAI as dependant variable). For model 1, TNF-α and IL17A SFCs were selected as predictor variables based on stepwise model selection. However, as the residual plots indicated a non-linear relationship in the data, non-linear transformation (log) of the predictor variables was carried out in the final model. Log transformed TNF-α SFCs and IL17A SFCs had a significant association and could independently predict DAS28 (see S3 File). Model 2 included IL17A SFCs and ESR as independent variables and had a statistically significant association with CDAI (see S4 File). Fig 4 visualizes the correlations between the CDAI, ESR, and IL17A SFCs in a 3D linear regression plot.

**Table 3:** Multiple linear regression to assess the association between disease activity (DAS 28 and CDAI) with cytokine expression and ESR.

Model	Outcome variable	Predictor variables	β	SE(β)	P-value of variables	R^2^	Adjusted R^2^	P-value of the model
1	DAS 28	TNF-α (Log)	0.420	0.175	0.0249	0.539	0.5031	4.3e-05
		IL17A(Log)	0.404	0.097	0.0003			
2	CDAI	IL17A	0.046	0.013	0.0014	0.439	0.3962	5.5e-04
		ESR	0.133	0.063	0.0458			

β, estimated coefficient; SE (β), estimated standard error of the β. P< 0.05 is statistically significant

## Discussion

Although many studies were conducted to assess the association of cytokines with RA disease activity, most of them were based on ELISA. Moreover, as some ELISA based findings on RA disease activity are contradicting, there is a need for testing the same hypothesis using different and more sensitive bioassays. In a study by Osiri et al., of the 81 RA patients whose serum were measured for 12 cytokines using ELISA, at least one of the cytokines were undetectable in 79 patients. Thus, the lower detection limit of cytokines by ELISA is a significant drawback in cytokine studies. This necessitates and justifies the use of more sensitive bioassays for cytokine studies in RA, such as ELISPOT.

The average symptom duration at the time of diagnosis and recruitment of the early RA patients’ group was as short as 4.75 months, possibly due to the use of ACR/EULAR 2010 criteria for diagnosis. It was observed that the number of IL-17A SFCs were higher in DMARDs naïve early RA patients compared to healthy controls (HC). Furthermore, when both early RA and established RA groups were combined, the number of IL17A SFCs and TNF-α SFCs (per 10^5^ PBMCs) were correlated with composite measures of RA disease activity, i.e. CDAI, and DAS28. Moreover, based on the multiple linear regression models constructed, log (IL17A SFCs) and log (TNF-α SFCs) could predict DAS28 score. Similarly, IL17A SFCs and ESR could predict CDAI score. These observations suggest that IL17A SFCs, among the four tested cytokines, has a higher potential of being a biomarker in RA.

As TNF-α and IL-17A are key pro-inflammatory cytokines in RA, current evidence shows that there is a systemic activation of the inflammatory cascade in early RA patients. Hence, the present study is also in line with previous studies [17, 29]. However, the number of PBMCs secreting these two cytokines in established RA patients was not significantly higher compared to control, although they had active disease status. The use of Methotrexate by the patients in established RA group may have led to the immunomodulation of the immune cells that drive the inflammation in RA. In addition to being an anti-proliferative agent, MTX inhibits T cell activation and suppresses the intercellular adhesion molecule expression by T cells, selectively down-regulates B cells, increases CD 95 sensitivity of activated T cells [30]. A study by Schotte et al. has shown that long-term exposure to biologic DMARDs such as Etanercept reduces the number of PBMCs that secrete TNF-α and IL-1β to healthy control levels in RA patients [31].

IL-17A SFCs showed the best correlation with clinical measures of disease activity in RA as it showed moderate correlations with CDAI, DAS28, swollen joint count, tender joint count, and joint pain-visual analog scale. Certain ELISA assay-based studies support these observations. A study by Metawi et al. showed elevated serum IL-17A levels in RA patients, which were in parallel with disease activity and severity as measured by several clinical markers [18]. Al-Saadani et al. showed the existence of a significant correlation between Th17 cells and serum IL-17 with DAS-28, ESR, CRP, and TNF-α [14]. However, a study by Osiri et al. did not demonstrate a statistically significant correlation between serum IL-17A levels and variables such as DAS 28, ESR, CRP, tender joint count, swollen joint count [19]. Therefore, it appears that there are contradictions between ELISA based studies for IL-17A in RA patients. Even though correlation does not imply causation, according to the current study, having a significant correlation with several key clinical measures of disease activity in RA, IL-17A shows its applicability as a potential biomarker in RA. Therefore, this warrants further studies.

Furthermore, there were moderate correlations between the number of TNF-α SFCs with DAS 28 and joint pain-VAS. A study by Jeng-Hsien Yen et al. have shown that there is a statistically significant positive correlation of serum TNF-α with the duration of morning stiffness, tender joint count, the Ritchie articular index, and ESR [32]. However, a study by Osiri et al. concluded that there are no statistically significant correlations between serum TNF-α and DAS28. Thus, it appears that there are discrepancies in the observations made between these studies. In the current study, IL-1β SFCs did not correlate with any of the clinical markers of disease activity. However, serum IL-1β levels reported to be positively correlated with Ritchie joint index, pain scores, and ESR and negatively correlated with haemoglobin concentration [33]. Moreover, serum IL-1β levels found to be positively correlated with the number of eroded joints in RA [34]. Similar to IL-1β in this study, the numbers of IL-10 SFCs were not correlated with ESR or clinical marker of disease activity in RA except a weak correlation with the swollen joint count. In line with the current study findings, a previous study by Cush et al. showed that the serum levels of IL-10 do not correlate with standard measures of clinical disease activity [35]. The balance between pro-inflammatory and anti-inflammatory cytokine response shifts towards the pro-inflammatory side in RA. IL-10, an anti-inflammatory mediator of inflammation, is secreted in active RA as well as an attempt to counteract the pro-inflammatory response. Schotte et al. showed a statistically significant negative correlation between IL-10 secretion by PBMCs and swollen joint count, indicating a potential protective role of IL-10 secretion against joint swelling in RA [31].

Multiple linear regression models were constructed to assess the predictability of CDAI and DAS 28 scores, using the number of SFCs of four cytokines tested and ESR. Thus, those regression analyses revealed that log-transformed TNF-α SFCs and log-transformed IL-17A SFCs are associated with and predictive of DAS 28 score. Likewise, IL-17A and ESR were associated with and predictive of CDAI score. The significant association of IL-17A with CDAI and DAS 28 scores, as observed in the 2 models, further supports the potential applicability of IL-17A as a biomarker for assessing the disease activity in RA.

The main practical limitation of the ELISPOT assay, being a staged procedure having lengthy steps such as overnight incubation with antibodies, is that it requires a couple of days to complete. This would make it challenging, if it is going to be used in the clinical setting. Furthermore, due to the dependency on PBMCs isolated from blood, assay performance can be affected by the recovery and handling of cells. A couple of limitations of the study are also acknowledged. Firstly, the use of manual counting rather than the use of an automated ELISPOT reader may have affected the results. Multiple assays were performed in the current study to analyze the samples; thus, inter-assay variability may have affected the results. However, this was unavoidable due to the analysis of fresh samples rather than cryopreserved and thawed sample. Moreover, such multiple assays facilitated the process of manual ELISPOT counting due to reduced workload during a given assay.

## Conclusion

The number of PBMCs secreting IL-17A is higher in DMARDs naïve early RA patients, but not in the established RA patients, compared to healthy controls. The number of IL-17A SFCs was correlated to 5 clinical markers of disease activity, i.e., DAS28, CDAI, joint pain-VAS, swollen and tender joint counts. Moreover, multivariate regression models showed that IL-17A SFCs is an important predictor of both DAS28 and CDAI. Hence, IL-17A SFCs has potential applicability as a biomarker of disease activity of RA.

## Supporting information

S1 File Dataset used for analysis. (XLSX)

S2 File R Markdown file of the correlogram (Fig 3). (HTML)

S3 File R markdown file of multiple linear regression model 1. (HTML)

S4 File R markdown file of multiple linear regression model 2. (HTML)
