# Supplementary material for: Potential applicability of cytokines as biomarkers of disease activity in rheumatoid arthritis: Enzyme-linked immunosorbent spot assay-based evaluation of TNF-α, IL-1β, IL-10 and IL-17A
Source: PLoS One. 2021 Jan 26;16(1):e0246111. doi: 10.1371/journal.pone.0246111 (PMC7837465; doi:10.1371/journal.pone.0246111)
Supplement: S2 File — (HTML) [file pone.0246111.s002.html]

Correlogram


# Correlogram

#### Keerthie Dissanayake

#### 9/7/2020

```
library(readxl)
ELISPOT <- read_excel("E:/Elispot/CORRELOGRAM/ELISPOT.xlsx")
summary(ELISPOT)
```

```
##       TNFa           IL-1ß             IL-10            IL-17A     
##  Min.   : 1680   Min.   :   93.4   Min.   :  26.0   Min.   :  1.0  
##  1st Qu.: 6200   1st Qu.: 1300.0   1st Qu.: 266.0   1st Qu.: 49.0  
##  Median :10933   Median : 4400.0   Median : 445.0   Median :179.0  
##  Mean   :11142   Mean   : 6828.9   Mean   : 552.6   Mean   :146.2  
##  3rd Qu.:15360   3rd Qu.: 7095.0   3rd Qu.: 767.0   3rd Qu.:208.0  
##  Max.   :23866   Max.   :47666.0   Max.   :2080.0   Max.   :321.0  
##       ESR            DAS 28           CDAI          Pain-VAS    
##  Min.   :18.00   Min.   :2.720   Min.   : 1.00   Min.   :1.000  
##  1st Qu.:32.00   1st Qu.:4.420   1st Qu.:12.00   1st Qu.:3.000  
##  Median :43.00   Median :4.940   Median :17.00   Median :5.000  
##  Mean   :47.55   Mean   :4.892   Mean   :16.69   Mean   :4.552  
##  3rd Qu.:61.00   3rd Qu.:5.400   3rd Qu.:20.00   3rd Qu.:6.000  
##  Max.   :98.00   Max.   :7.190   Max.   :43.00   Max.   :7.000  
##       TJC              SJC      
##  Min.   : 0.000   Min.   :0.00  
##  1st Qu.: 2.000   1st Qu.:1.00  
##  Median : 4.000   Median :2.00  
##  Mean   : 5.897   Mean   :2.31  
##  3rd Qu.: 7.000   3rd Qu.:3.00  
##  Max.   :26.000   Max.   :8.00
```

```
library(corrplot)
```

```
## corrplot 0.84 loaded
```

```
M <- cor(ELISPOT)

library(RColorBrewer)
cor.mtest <- function(mat, ...) {
  mat <- as.matrix(mat)
  n <- ncol(mat)
  p.mat<- matrix(NA, n, n)
  diag(p.mat) <- 0
  for (i in 1:(n - 1)) {
    for (j in (i + 1):n) {
      tmp <- cor.test(mat[, i], mat[, j], ...)
      p.mat[i, j] <- p.mat[j, i] <- tmp$p.value
    }
  }
  colnames(p.mat) <- rownames(p.mat) <- colnames(mat)
  p.mat
}
p.mat <- cor.mtest(ELISPOT)
head(p.mat[, 1:5])
```

```
##                TNFa        IL-1ß       IL-10      IL-17A         ESR
## TNFa   0.0000000000 0.0002367196 0.020668973 0.135520726 0.162580784
## IL-1ß  0.0002367196 0.0000000000 0.009565294 0.113701544 0.730272340
## IL-10  0.0206689726 0.0095652943 0.000000000 0.274054019 0.158539681
## IL-17A 0.1355207258 0.1137015438 0.274054019 0.000000000 0.380155302
## ESR    0.1625807836 0.7302723400 0.158539681 0.380155302 0.000000000
## DAS 28 0.0155433451 0.2364921242 0.386686711 0.001068341 0.001060558
```

```
col <- colorRampPalette(c("#BB4444", "#EE9988", "#FFFFFF", "#77AADD", "#4477AA"))
corrplot(M, method="color", col=col(200),
         type="upper", order="hclust",
         addCoef.col = "black",
         tl.col="black", tl.srt=45,
         p.mat = p.mat, sig.level = 0.05, insig = "blank",
         diag=FALSE
)
```
