# Supplementary material for: Potential applicability of cytokines as biomarkers of disease activity in rheumatoid arthritis: Enzyme-linked immunosorbent spot assay-based evaluation of TNF-α, IL-1β, IL-10 and IL-17A
Source: PLoS One. 2021 Jan 26;16(1):e0246111. doi: 10.1371/journal.pone.0246111 (PMC7837465; doi:10.1371/journal.pone.0246111)
Supplement: S3 File — (HTML) [file pone.0246111.s003.html]

DAS28\_MLR


# DAS28\_MLR

#### Keerthie Dissanayake

#### 9/7/2020

```
library(readxl)
DAS_MLR <- read_excel("E:/Elispot/Multiple linear regreassion/FINALDASMODEL/DAS_MLR.xlsx")
summary(DAS_MLR)
```

```
##       TNFa            IL1b              IL10            IL17A      
##  Min.   : 1680   Min.   :   93.4   Min.   :  26.0   Min.   :  1.0  
##  1st Qu.: 6200   1st Qu.: 1300.0   1st Qu.: 266.0   1st Qu.: 49.0  
##  Median :10933   Median : 4400.0   Median : 445.0   Median :179.0  
##  Mean   :11142   Mean   : 6828.9   Mean   : 552.6   Mean   :146.2  
##  3rd Qu.:15360   3rd Qu.: 7095.0   3rd Qu.: 767.0   3rd Qu.:208.0  
##  Max.   :23866   Max.   :47666.0   Max.   :2080.0   Max.   :321.0  
##       DAS       
##  Min.   :2.720  
##  1st Qu.:4.420  
##  Median :4.940  
##  Mean   :4.892  
##  3rd Qu.:5.400  
##  Max.   :7.190
```

```
#package MASS
library(MASS)
# Fit the full model 
REG1 <- lm(DAS ~., data = DAS_MLR)
REG1
```

```
## 
## Call:
## lm(formula = DAS ~ ., data = DAS_MLR)
## 
## Coefficients:
## (Intercept)         TNFa         IL1b         IL10        IL17A  
##   3.560e+00    6.749e-05   -1.929e-05   -9.001e-05    5.214e-03
```

```
# Stepwise regression model
step.model <- stepAIC(REG1, direction = "both", 
                      trace = FALSE)
summary(step.model)
```

```
## 
## Call:
## lm(formula = DAS ~ TNFa + IL17A, data = DAS_MLR)
## 
## Residuals:
##      Min       1Q   Median       3Q      Max 
## -1.29665 -0.59509 -0.08282  0.38472  2.06179 
## 
## Coefficients:
##              Estimate Std. Error t value Pr(>|t|)    
## (Intercept) 3.635e+00  3.375e-01  10.771 4.41e-11 ***
## TNFa        4.810e-05  2.451e-05   1.962  0.06049 .  
## IL17A       4.936e-03  1.573e-03   3.137  0.00421 ** 
## ---
## Signif. codes:  0 '***' 0.001 '**' 0.01 '*' 0.05 '.' 0.1 ' ' 1
## 
## Residual standard error: 0.8 on 26 degrees of freedom
## Multiple R-squared:  0.4183, Adjusted R-squared:  0.3735 
## F-statistic: 9.348 on 2 and 26 DF,  p-value: 0.0008734
```

```
# testing for linear regression assumptions
par(mfrow = c(2, 2))
plot(REG1)
```

```
#Independant variables were limited to 2 based on the previous model and sample number
REG2=lm(DAS~TNFa+IL17A,data=DAS_MLR)
summary(REG2)
```

```
## 
## Call:
## lm(formula = DAS ~ TNFa + IL17A, data = DAS_MLR)
## 
## Residuals:
##      Min       1Q   Median       3Q      Max 
## -1.29665 -0.59509 -0.08282  0.38472  2.06179 
## 
## Coefficients:
##              Estimate Std. Error t value Pr(>|t|)    
## (Intercept) 3.635e+00  3.375e-01  10.771 4.41e-11 ***
## TNFa        4.810e-05  2.451e-05   1.962  0.06049 .  
## IL17A       4.936e-03  1.573e-03   3.137  0.00421 ** 
## ---
## Signif. codes:  0 '***' 0.001 '**' 0.01 '*' 0.05 '.' 0.1 ' ' 1
## 
## Residual standard error: 0.8 on 26 degrees of freedom
## Multiple R-squared:  0.4183, Adjusted R-squared:  0.3735 
## F-statistic: 9.348 on 2 and 26 DF,  p-value: 0.0008734
```

```
par(mfrow = c(2, 2))
plot(REG2)
```

```
#Independant variables were Log transformed 
REG3=lm(DAS~log(TNFa)+log(IL17A),data=DAS_MLR)
summary(REG3)
```

```
## 
## Call:
## lm(formula = DAS ~ log(TNFa) + log(IL17A), data = DAS_MLR)
## 
## Residuals:
##     Min      1Q  Median      3Q     Max 
## -1.1360 -0.3927 -0.2260  0.2717  1.8690 
## 
## Coefficients:
##             Estimate Std. Error t value Pr(>|t|)    
## (Intercept) -0.71939    1.54350  -0.466 0.645040    
## log(TNFa)    0.42039    0.17520   2.399 0.023877 *  
## log(IL17A)   0.40374    0.09668   4.176 0.000295 ***
## ---
## Signif. codes:  0 '***' 0.001 '**' 0.01 '*' 0.05 '.' 0.1 ' ' 1
## 
## Residual standard error: 0.7125 on 26 degrees of freedom
## Multiple R-squared:  0.5386, Adjusted R-squared:  0.5031 
## F-statistic: 15.17 on 2 and 26 DF,  p-value: 4.298e-05
```

```
par(mfrow = c(2, 2))
plot(REG3)
```

```
#testing for multicolinearity
car::vif(REG3)
```

```
##  log(TNFa) log(IL17A) 
##   1.070676   1.070676
```
