# Supplementary material for: Potential applicability of cytokines as biomarkers of disease activity in rheumatoid arthritis: Enzyme-linked immunosorbent spot assay-based evaluation of TNF-α, IL-1β, IL-10 and IL-17A
Source: PLoS One. 2021 Jan 26;16(1):e0246111. doi: 10.1371/journal.pone.0246111 (PMC7837465; doi:10.1371/journal.pone.0246111)
Supplement: S4 File — (HTML) [file pone.0246111.s004.html]

CDAI\_MLR


# CDAI\_MLR

#### Keerthie Dissanayake

#### 9/7/2020

```
library(readxl)
CDAI_MLR <- read_excel("E:/Elispot/Multiple linear regreassion/FINALCDAIMODEL/CDAI_MLR.xlsx")
View(CDAI_MLR)
attach(CDAI_MLR)
summary(CDAI_MLR)
```

```
##       TNFa            IL1b              IL10            IL17A      
##  Min.   : 1680   Min.   :   93.4   Min.   :  26.0   Min.   :  1.0  
##  1st Qu.: 6200   1st Qu.: 1300.0   1st Qu.: 266.0   1st Qu.: 49.0  
##  Median :10933   Median : 4400.0   Median : 445.0   Median :179.0  
##  Mean   :11142   Mean   : 6828.9   Mean   : 552.6   Mean   :146.2  
##  3rd Qu.:15360   3rd Qu.: 7095.0   3rd Qu.: 767.0   3rd Qu.:208.0  
##  Max.   :23866   Max.   :47666.0   Max.   :2080.0   Max.   :321.0  
##       ESR             CDAI      
##  Min.   :18.00   Min.   : 1.00  
##  1st Qu.:32.00   1st Qu.:12.00  
##  Median :43.00   Median :17.00  
##  Mean   :47.55   Mean   :16.69  
##  3rd Qu.:61.00   3rd Qu.:20.00  
##  Max.   :98.00   Max.   :43.00
```

```
#package MASS 
library(MASS)

# Fit the full model 
REG1 <- lm(CDAI ~., data = CDAI_MLR)
REG1
```

```
## 
## Call:
## lm(formula = CDAI ~ ., data = CDAI_MLR)
## 
## Coefficients:
## (Intercept)         TNFa         IL1b         IL10        IL17A          ESR  
##   2.762e+00    3.366e-04   -4.612e-05   -4.047e-03    4.467e-02    1.304e-01
```

```
par(mfrow = c(2, 2))
plot(REG1)
```

```
# Stepwise regression model

step.model <- stepAIC(REG1, direction = "both", 
                trace = FALSE)
summary(step.model)
```

```
## 
## Call:
## lm(formula = CDAI ~ TNFa + IL10 + IL17A + ESR, data = CDAI_MLR)
## 
## Residuals:
##    Min     1Q Median     3Q    Max 
## -8.182 -4.310 -1.314  2.730 19.370 
## 
## Coefficients:
##               Estimate Std. Error t value Pr(>|t|)   
## (Intercept)  2.8973286  3.6146472   0.802   0.4307   
## TNFa         0.0003008  0.0002210   1.361   0.1862   
## IL10        -0.0043072  0.0031259  -1.378   0.1809   
## IL17A        0.0441319  0.0130103   3.392   0.0024 **
## ESR          0.1339554  0.0649659   2.062   0.0502 . 
## ---
## Signif. codes:  0 '***' 0.001 '**' 0.01 '*' 0.05 '.' 0.1 ' ' 1
## 
## Residual standard error: 6.557 on 24 degrees of freedom
## Multiple R-squared:  0.4973, Adjusted R-squared:  0.4135 
## F-statistic: 5.935 on 4 and 24 DF,  p-value: 0.001816
```

```
# model based on selected independant variables (decided by stepwise model and sample number)

REG2 <- lm(CDAI ~IL17A+ESR,data = CDAI_MLR)
summary(REG2)
```

```
## 
## Call:
## lm(formula = CDAI ~ IL17A + ESR, data = CDAI_MLR)
## 
## Residuals:
##    Min     1Q Median     3Q    Max 
## -8.451 -4.897 -1.600  2.878 21.327 
## 
## Coefficients:
##             Estimate Std. Error t value Pr(>|t|)   
## (Intercept)  3.71612    3.48273   1.067  0.29577   
## IL17A        0.04562    0.01273   3.584  0.00137 **
## ESR          0.13257    0.06321   2.097  0.04583 * 
## ---
## Signif. codes:  0 '***' 0.001 '**' 0.01 '*' 0.05 '.' 0.1 ' ' 1
## 
## Residual standard error: 6.652 on 26 degrees of freedom
## Multiple R-squared:  0.4393, Adjusted R-squared:  0.3962 
## F-statistic: 10.19 on 2 and 26 DF,  p-value: 0.0005409
```

```
par(mfrow = c(2, 2))
plot(REG2)
```

```
#testing the multicolinearity
car::vif(REG2)
```

```
##    IL17A      ESR 
## 1.029484 1.029484
```
